# Strain-specific antiviral activity of iminosugars against human influenza A viruses

**DOI:** 10.1093/jac/dku349

**Published:** 2014-09-15

**Authors:** S. Hussain, J. L. Miller, D. J. Harvey, Y. Gu, P. B. Rosenthal, N. Zitzmann, J. W. McCauley

**Affiliations:** 1 Division of Virology, Medical Research Council National Institute for Medical Research, Mill Hill, London NW7 1AA, UK; 2 Division of Physical Biochemistry, Medical Research Council National Institute for Medical Research, Mill Hill, London NW7 1AA, UK; 3 Oxford Glycobiology Institute, Department of Biochemistry, University of Oxford, South Parks Road, Oxford OX1 3QU, UK; 4 Department of Biological Sciences, Gibbet Hill Campus, University of Warwick, Coventry CV4 7AL, UK; 5 Confocal Imaging and Analysis Laboratory, Medical Research Council National Institute for Medical Research, Mill Hill, London NW7 1AA, UK

**Keywords:** *N*-butyl-deoxynojirimycin, *N*B-DNJ, *N*-nonyl-deoxynojirimycin, *N*N-DNJ, *N*-nonyl-deoxygalactojirimycin, *N*N-DGJ

## Abstract

**Objectives:**

Drugs that target host cell processes can be employed to complement drugs that specifically target viruses, and iminosugar compounds that inhibit host α-glucosidases have been reported to show antiviral activity against multiple viruses. Here the effect and mechanism of two iminosugar α-glucosidase inhibitors, *N*-butyl-deoxynojirimycin (*N*B-DNJ) and *N*-nonyl-deoxynojirimycin (*N*N-DNJ), on human influenza A viruses was examined.

**Methods:**

The viruses examined were a recently circulating seasonal influenza A(H3N2) virus strain A/Brisbane/10/2007, an older H3N2 strain A/Udorn/307/72, and A/Lviv/N6/2009, a strain representative of the currently circulating pandemic influenza A(H1N1)pdm09 virus.

**Results:**

The inhibitors had the strongest effect on Brisbane/10 and *N*N-DNJ was more potent than *N*B-DNJ. Both compounds showed antiviral activity in cell culture against three human influenza A viruses in a strain-specific manner. Consistent with its action as an α-glucosidase inhibitor, *N*N-DNJ treatment resulted in an altered glycan processing of influenza haemagglutinin (HA) and neuraminidase (NA), confirmed by MS. *N*N-DNJ treatment was found to reduce the cell surface expression of the H3 subtype HA. The level of sialidase activity of NA was reduced in infected cells, but the addition of exogenous sialidase to the cells did not complement the *N*N-DNJ-mediated inhibition of virus replication. Using reassortant viruses, the drug susceptibility profile was determined to correlate with the origin of the HA.

**Conclusions:**

*N*N-DNJ inhibits influenza A virus replication in a strain-specific manner that is dependent on the HA.

## Introduction

Influenza A viruses and influenza B viruses cause seasonal human influenza but influenza A viruses pose an additional risk of zoonotic infection, with the potential of a host switch and the generation of pandemic influenza. Influenza epidemics occur almost every year as the virus undergoes antigenic drift but pandemics are rare and have occurred with the H1N1 (1918, 1977 and 2009), H2N2 (1957) and H3N2 (1968) subtype viruses. The 1918 ‘Spanish flu’ pandemic was by the far the most severe resulting in an estimated 40–100 million deaths worldwide,^1^ while the 2009 swine flu pandemic caused an estimated 200 000 deaths worldwide.^2^

Influenza viruses encode two glycoproteins, haemagglutinin (HA) and neuraminidase (NA), and a transmembrane ion channel M2. Both glycoproteins play important roles in the virus life cycle. HA mediates viral attachment via sialic acid receptors on the surface of a cell and the subsequent fusion of the viral and endosomal membranes (reviewed in Skehel and Wiley^3^). NA, a sialidase, releases progeny virus particles from infected cells by removing sialic acids from the host cell and the virus itself.^4–6^

The current measures against human influenza involve vaccination and the use of antiviral drugs. The effectiveness of vaccination is limited by the continuous antigenic drift of the virus as it evolves, and at the onset of a pandemic there is unlikely to be an effective vaccine available. Therefore, antiviral drugs can play an important role in mitigating the effects of influenza. There are two widely licensed drug classes, the M2 inhibitors (adamantanes) and NA inhibitors (oseltamivir and zanamivir). The M2 inhibitors are currently not recommended for the treatment of influenza due to widespread resistance of the circulating human strains of virus.^7,8^ There are many examples of the emergence of virus strains resistant to NA inhibitors in several subtypes, including examples of resistance of H1N1pdm09 viruses, H5N1 viruses and the newly emerged H7N9 viruses. Importantly, resistance to oseltamivir became widespread in previously circulating human H1N1 viruses from 2007 until the extinction of these strains in 2009 and the emergence of the H1N1pdm09 virus.^9,10^ Drugs that act as antivirals by targeting host cell processes may lower the risk of drug resistance in a virus and make an attractive strategy for antiviral therapy, particularly in combination therapy with drugs specifically targeting viral proteins.

The need to develop novel anti-influenza therapies led us to test the antiviral activity of two iminosugar compounds, *N*-butyl-deoxynojirimycin (*N*B-DNJ) and *N*-nonyl-deoxynojirimycin (*N*N-DNJ), against human influenza A viruses. These glucose analogue compounds can act as inhibitors of α-glucosidases in the endoplasmic reticulum (ER) (reviewed in Dwek *et al.*^11^); in addition, DNJ derivatives with a minimum alkyl chain length of at least three carbons can inhibit ceramide-specific glucosyltransferases (CerGlcT), thus inhibiting glucosphingolipid (GSL) synthesis.^11^

The antiviral potential of iminosugars has been explored for human HIV,^12,13^ hepatitis B,^14,15^ hepatitis C (HCV),^16,17^ bovine viral diarrhoea virus (BVDV)^18,19^ and the flaviviruses dengue virus and West Nile virus.^20,21^ Moreover, long alkyl DNJ derivatives such as *N*N-DNJ also exhibit antiviral effects associated with the alkyl side chain, such as inhibition of the p7 ion channel of HCV in a genotype-dependent manner.^22,23^

Many glycoproteins require N-linked glycosylation for folding and maturation. Nascent N-linked glycoproteins entering the ER are glycosylated with a high-mannose oligosaccharide precursor rich in terminal glucose residues that are sequentially removed by α-glucosidases. Subsequent α-mannosidase activity and further processing can occur (reviewed in Dwek *et al.*^11^). The inhibition of α-glucosidases by *N*B-DNJ and *N*N-DNJ hinders the formation of monoglucosylated glycans, thus preventing interaction with the glycan-binding proteins calnexin and calreticulin (which mediate protein folding), and so preventing correct protein folding and glycan modification.

The ER chaperones calnexin and calreticulin mediate the proper cotranslational folding of both influenza HA and NA^24–30^ so this interaction is potentially susceptible to inhibition. Treatment with glucosidase inhibitors inhibits the interaction of X-31 H3 subtype HA with calnexin and calreticulin^24,25,27,28^ and similarly the interaction of N9 NA with calnexin and calreticulin is also inhibited.^29^ However, considerable variation in the antiviral effects of α-glucosidase inhibitors against influenza A viruses has been observed in different studies. The release of infectious particles of the influenza A laboratory-adapted H1N1 strains A/Puerto Rico/8/34 (PR8) and A/NWS/33 (NWS) in MDCK cells treated with castanospermine (CST),^31,32^ PR8 in chick embryo cells treated with bromoconduritol (BC)^33^ and the avian influenza virus Rostock (H7N1) in chick embryo cells treated with *N*-methyl-deoxynojirimycin (*N*M-DNJ)^34^ was not significantly reduced when compared with untreated cells. However, a reduction in the release of Rostock from chick embryo cells on BC treatment,^33^ and of a reassortant NWS-N8 virus [which had the HA (H1) of NWS and the NA (N8) of A/duck/Ukraine/1/63] from MDCK cells on CST treatment was observed.^35^ These studies suggest strain-dependent antiviral effects of α-glucosidase inhibitors on influenza viruses.

Since previous studies have determined antiviral activity against well-established laboratory strains of influenza A viruses, we tested more recent viruses for the antiviral effects of iminosugars. *N*B-DNJ has been used in the clinic for over 10 years for the treatment of Gaucher's disease without any serious adverse effects, and *N*N-DNJ, which has a longer alkyl chain than the latter, is potentially a more potent antiviral derivative. Here, we show that *N*B-DNJ and *N*N-DNJ exhibit antiviral effects against two viruses of the influenza A(H3N2) subtype, A/Udorn/307/72 (Udorn) and A/Brisbane/10/2007 (Brisbane/10), and a virus that is a representative of the A(H1N1)pdm09 virus, A/Lviv/N6/2009 (Lviv). Our studies show that *N*B-DNJ and *N*N-DNJ inhibit virus replication in a strain-specific manner, which is likely to be dependent on the origin of the HA. The inhibition of α-glucosidases in cells alters the glycosylation pattern of the virus glycoproteins synthesized in the cells, reducing the surface transport of HA and the sialidase activity of NA.

## Materials and methods

### Cells, viruses and inhibitors

Canine kidney MDCK II cells came from Phillips-University Marburg, Germany and were cultured in MEM (Sigma) containing 10% (v/v) FBS (Perbio), 100 U/mL penicillin/streptomycin (Sigma) and 100 U/mL GlutaMAX (Invitrogen). Infection was carried out in serum-free MEM containing 100 U/mL penicillin/streptomycin (Sigma), 100 U/mL GlutaMAX (Invitrogen) and 0.14% (w/v) BSA (Sigma). Influenza A viruses Brisbane/10 (H3N2), Udorn (H3N2) and Lviv (H1N1) were egg-passaged isolates obtained from stocks held by the WHO Collaborating Centre for Reference and Research on Influenza (WHO CC) at the National Institute for Medical Research (NIMR), Mill Hill, UK. High-growth reassortant viruses X-171b (H3N2) and X-181 (H1N1) were kindly provided by the National Institute for Biological Standards and Control (NIBSC), Hertfordshire, UK. The iminosugar compounds *N*B-DNJ, *N*N-DNJ and *N*-nonyl-deoxygalactojirimycin (*N*N-DGJ) were kindly provided by Oxford GlycoSciences, UK and stocks were made with DMSO as a solvent. Zanamivir was obtained from GlaxoSmithKline. CPNA, a neuraminidase from *Clostridium perfringens*, was obtained from Roche.

### Antibodies and sera

The primary antibodies used were as follows: SH487 sheep serum anti-HA of Brisbane/10 and SH510 sheep serum anti-HA of A/California/07/2009 (both used at 1: 1000 for western blot and 1: 100 for immunofluorescence) were a kind gift from Mr Robert Newman (NIBSC); PE78 mouse monoclonal anti-NA of A/California/04/2009 antibody (used at 1: 100 for immunofluorescence) was a kind gift from Dr Richard Webby (St Jude Children's Research Hospital, Memphis, USA); R10/8 rabbit serum anti-X-31 (used at 1: 100 for immunofluorescence) was obtained from the WHO CC NIMR; mouse monoclonal anti-nucleoprotein (NP) antibody (ab20343, AbCam) was diluted at 1: 1000 for immunostaining virus plaques. Secondary antibodies used were as follows: goat anti-rabbit Alexa Fluor 488, donkey anti-sheep Alexa Fluor 488 and goat anti-mouse Alexa Fluor 594 (all from Invitrogen) were used at a dilution of 1: 1000. Goat anti-mouse horse radish peroxidase conjugate antibody (Bio-Rad, 172-1011) was used at a dilution of 1: 1000 for immunostaining plaques. Rabbit anti-sheep Dylight 800 antibody was used at 1: 5000 for western blotting. Ferret sera for HA inhibition (HI) assays, ferret anti- Brisbane/10 and ferret anti-Lviv, both receptor-destroying-enzyme treated, were obtained from the WHO CC NIMR.

### Primers

All primers used in this study were obtained from the WHO CC NIMR. Primer sequences are available as Supplementary data at *JAC* Online.

### Generation and characterization of reassortant viruses

Confluent monolayers of MDCK cells were infected at an moi of 10 pfu/cell with different ratios of the two viruses Brisbane/10 and Lviv, essentially as described by Baigent *et al*.^36^ The subtype of HA was determined by HI assays with ferret serum raised against Brisbane/10 or Lviv. The subtype of NA was determined following isolation of RNA from the virus in allantoic fluid and amplification of the genomic segment in a one-step RT–PCR with N1- and N2-specific primers. All genomic segments of the reassortant H3N1 virus were identified by nucleotide sequence analysis.

### RNA extraction, RT–PCR and sequence analysis

All viral RNA extractions were performed using the QIAamp Viral RNA Mini Kit (Qiagen) using on-column DNase digestion (Qiagen). Reverse transcription was performed with the Uni12 primer using the Verso cDNA kit (Thermo Scientific). PCR reactions were performed using Pfu Ultra II fusion HS polymerase (Stratagene #600674-51) according to the manufacturer's protocol. PCR products were purified for sequencing by Illustra GFX PCR DNA and Gel Band Purification kit (GE Healthcare). Sequencing reactions were performed using BigDye^®^ Terminator v1.2 Cycle Sequencing Kit (Applied Biosystems) (according to the manufacturer's instructions) and samples were run on an ABI 3730xI DNA Analyser.

### Cell viability assay

Confluent monolayers of MDCK cells were treated with increasing concentrations of *N*B-DNJ or *N*N-DNJ and equivalent amounts of DMSO, serving as a control, in serum-free medium for 48 h. Cells were subsequently treated with CellTiter-Blue™ (Promega) reagent according to the manufacturer's protocol and fluorescence was measured (560_Ex_/590_Em_). As a positive control for cytotoxicity, cells were treated with digitonin (0.5 mg/mL; Sigma) for 10 min before the addition of CellTiter-Blue™ reagent. The CC_50_ and CC_90_ were calculated for each experiment by linear interpolation.

### Virus titration

#### Plaque assay

Confluent monolayers of MDCK cells were infected with 10-fold serial dilutions of virus and plaque assays were performed using Avicel overlay medium, essentially as described by Matrosovich *et al.*^37^ Cells were fixed and stained at various times post-infection (pi) with 4% (v/v) formaldehyde and 0.2% (w/v) toluidine blue in PBS, or the plaques were immunostained for influenza NP as described by Sullivan *et al.*^38^ Plaque numbers were scored and plaque size was measured to the nearest 0.1 mm using a PEAK™ Scale Lupe (×10 magnification).

#### HA assay

HA assays were performed using 0.75% (v/v) turkey red blood cells/PBS as described in the WHO methods.^39^

### Virus yield assays and plaque reduction assays

#### Single-cycle high-moi virus yield assay

Confluent monolayers of MDCK cells were washed twice with PBS and infected at an moi of 10 pfu per cell for 1 h at room temperature. After removal of the inoculum, cells were washed four times with PBS, treated with the indicated concentrations of *N*B-DNJ and *N*N-DNJ in serum-free virus growth medium and incubated at 37°C, 5% CO_2_. A DMSO control corresponded to an amount of DMSO in the medium equivalent to that present at the highest concentration of the drug. At 3 h pi, the medium was removed, the cells were washed four times with PBS and the medium with the same concentrations of drug was replaced. The cells were then again incubated at 37°C, 5% CO_2_. At 20 h pi the virus yield from cell culture supernatants was measured by HA assays and by plaque assays performed in duplicate. Mean HA titres or infectious virus titres were calculated as a percentage of HA or infectious virus titres, respectively, from untreated cells for each drug treatment condition in an experiment. The IC_50_ and IC_90_ of viral growth were calculated for each experiment by linear interpolation. To determine whether the presence of *N*N-DNJ in the cell culture supernatants affected the infectious virus titres measured by plaque assay or HA titres in the HA assay, different concentrations of *N*N-DNJ were added to a fixed volume of each virus used in these studies. For plaque assays, MDCK cells were infected at an moi of 0.0001 pfu/cell with inoculum containing *N*N-DNJ or untreated inoculum and, after removal of the inoculum, overlaid with medium in the absence of drug. Plaques were assessed as described earlier. Similarly, HA assays were performed on viruses in the presence of the highest concentration of *N*N-DNJ present in the cell culture supernatants or a control lacking drug. The presence of *N*N-DNJ did not affect the measurements in either assay.

#### Multicycle plaque reduction assay

Confluent monolayers of MDCK cells were washed twice with PBS and infected at an moi of ∼0.0001 pfu per cell for 1 h at room temperature. After removal of the inoculum, the cells were treated with increasing concentrations of drug in the overlay containing trypsin. The DMSO control represents an amount of DMSO in the overlay equivalent to that present in the overlay with the highest concentration of drug used. At time ≥30 h pi, dependent on the strain, the overlay was removed, and the cells were fixed with formaldehyde and then stained with toluidine blue or immunostained for influenza NP. Plaque assays were performed in duplicate in each experiment. The mean plaque number as a percentage of the mean plaque number from the untreated cells was calculated for each drug condition in an experiment. To assess the effects of the treatments on plaque size, 30 plaques were measured (to the nearest 0.1 mm) per drug treatment condition using a PEAK Scale Lupe ×10. The median plaque size as a percentage of the median plaque size from the untreated cell control was calculated for each drug condition in an experiment. IC_50_ and IC_90_ values were calculated for each experiment by linear interpolation.

### Quantification of cells immunostained for influenza NP

Confluent monolayers of MDCK cells in 96-well plates were washed twice with PBS and then infected at an moi of 0.1 pfu per cell for 1 h at room temperature. After removal of the inoculum, the cells were washed twice with PBS and treated with 62.5 μM *N*N-DNJ or the equivalent DMSO control in serum-free medium. At 6 h pi the cells were fixed and immunostained for NP as described above. The 96-well plates were scanned with a house-assembled scanner using an IX70 Olympus microscope with a ×10 objective (numerical aperture = 0.3), using Surveyor software (Objective Imaging Ltd^®^). The images were quantified using the high-throughput quantification method described by Sullivan *et al.*^38^ NP-positive cells were counted based on their size in pixels, which was calibrated visually in advance. NP-positive cells from triplicate wells were averaged for each drug treatment in an experiment. Finally, the mean number of NP-positive cells from drug-treated cells as a percentage of mean NP-positive cells from untreated cells was calculated for each experiment.

### Purification of virus from cell culture supernatants

Confluent monolayers of MDCK cells were washed twice with PBS, infected at an moi of 10 pfu per cell for 1 h at room temperature and treated as in the virus yield assay. At 19 h pi for Brisbane/10 and Lviv, and 11 h pi for Udorn, the cell culture supernatants were harvested and clarified of cells by centrifugation, and virus was purified by ultracentrifugation at 157 000 **g** for 3 h at 4°C through a 30% (w/v) sucrose/PBS cushion. Virus proteins were separated by SDS-PAGE on 10% polyacrylamide gels under reducing conditions and protein bands were visualized by Coomassie blue staining. Gels were imaged using the scanner Epson perfection v750 pro and densitometry was performed using ImageJ software.

### qRT–PCR to detect virus purified from tissue culture supernatants

Cell culture supernatants from high-moi infections were harvested at 9 h pi and clarified, and virus was purified by ultracentrifugation as described above. Virus pellets were resuspended in PBS and RNA was extracted as described earlier. Reverse transcription was performed with Uni12 primer using the Omniscript RT kit (Qiagen). The qRT–PCR reactions were performed using TaqMan^®^ Fast Universal PCR Master Mix (Applied Biosystems) with primers and probes from the CDC to detect influenza virus Segment 7. Samples were run on Applied Biosystems 7500 Fast Real-Time PCR machine. qRT–PCR was performed in triplicate per sample and mock-infected-cell, no-RT and no-template controls were used in each experiment. C_T_ values from virus pellets from *N*N-DNJ-treated cells were subtracted from control cells infected with that virus (ΔC_T_) and Segment 7 expression from virus purified from *N*N-DNJ-treated cells was calculated as 2^−(ΔCT)^ and expressed as a percentage of the control (DMSO-treated cells).

### ^35^S-labelling of viral proteins

Cells were infected at an moi of 10 pfu per cell and at 6 h pi cells were labelled with 100 μCi/mL [^35^S]methionine/cysteine (EXPRESS ^35^S Protein Labelling Mix, Perkin-Elmer # NEG072002MC) in Met-Cys free medium in the presence of drug or DMSO for 40 min and analysed essentially as described by McCauley and Penn.^40^

### Immunofluorescence and quantification of protein expression

Cells were infected at an moi of 5 pfu per cell and at 8 h pi cells were fixed in 4% (w/v) paraformaldehyde in PBS for 20 min at room temperature and then washed three times with PBS. Cells were immunostained with virus strain-specific primary Ab raised against HA or NA diluted in 0.35% BSA (w/v) in PBS for 1 h at room temperature and were washed three times with PBS and stained with Alexa Fluor 488- or 594-conjugated secondary Ab diluted in 0.35% BSA (w/v) in PBS for 1 h at 4°C; they were then washed three times with PBS. Cells were stained with 0.35 μg/mL DAPI and mounted onto slides in Prolong^®^ Gold antifade reagent (Invitrogen).

Images were acquired on a DeltaVision RT microscope (Applied Precision Ltd, USA) using a ×100 objective (numerical aperture = 1.4) onto a CoolSNAP HQ CCD camera (Photometrics Ltd) obtaining Z-sections of 0.2 μm to a depth of 7 μm with appropriate filters and deconvolved using the softWoRx software (AppliedPrecision^®^). Then 16-bit 2D maximal intensity projections were generated using Fiji software. The overall fluorescence intensity above the background for each infected cell was determined and the area of the cell was measured. The overall fluorescence intensity for each cell is plotted as number of fluorescent pixels per μm^2^.

### Sialidase activity of NA from post-nuclear supernatants

Confluent monolayers of MDCK cells, infected at an moi of 5 pfu per cell, were subsequently harvested on ice, washed in 1 mM EDTA in PBS and removed with a cell scraper, followed by centrifugation for 10 min at 1200 **g** at 4°C. The pellets were resuspended in isotonic buffer (20 mM Tris-HCl pH 7.4, 140 mM NaCl), at the same volume as the pellet, and a 20-fold volume of hypotonic buffer [20 mM Tris-HCl pH 7.4, with PI (Sigma P8340) diluted 1: 100] was added. Cells were sheared by passing them 15 times through a 21 gauge needle and five times through a 27 gauge needle and nuclei were pelleted by centrifugation at 150 **g** for 5 min at 4°C. Protein concentrations from post-nuclear supernatants were normalized by quantification with a Bradford assay (Bio-Rad) prior to a sialidase assay using 2′-(4-methylumbelliferyl)-α-D-*N*-acetylneuraminic acid (MuNANA).^39^

### MS analysis of glycans of glycoproteins from purified virus

Virus from cell culture supernatants was purified by ultracentrifugation as described earlier and the virus proteins were separated by SDS-PAGE under reducing conditions. The influenza glycoprotein bands were confirmed by MS. In brief, this involved the excision of Coomassie blue-stained bands corresponding to the molecular weights of HA and NA, and the reduction, alkylation and removal of N-linked glycans by in-gel digestion with peptide-*N*-glycosidase F (PNGase F; Roche Life Sciences). Peptide fragments were generated by in-gel digestion with chymotrypsin, trypsin and elastase. Digested protein acidified to a final concentration of 0.1% trifluoroacetic acid was loaded onto an ultimate 3000 nanoRSLC (rapid separation LC) HPLC using a stepwise gradient of acetonitrile and 0.1% formic acid. Eluant was introduced into a Linear Trap Quadrupole Orbitrap Velos Pro (Thermo Scientific) using a Proxeon nano-electrospray source (Thermo Scientific). Subsequent to tandem MS, proteins were identified using the search engine Mascot 2.4 (Matrix Science, UK) and the data were searched against a custom database consisting of the sequences obtained by the sequencing of HA and NA from the RT–PCR products of viral RNA.

For the MS analysis of the glycans in the glycoproteins, Coomassie blue-stained SDS-PAGE gel bands of influenza glycoproteins HA and NA were excised, washed five times alternately with acetonitrile and deionized water and rehydrated with a 3000 U/mL of aqueous PNGase F (New England Biolabs, Ipswich, USA) solution in gel, as described by Küster *et al*.^41^ The gel bands were subsequently incubated at 37°C for 12 h and the enzymatically released glycans were eluted with water. Samples were diluted into 3 µL of H_2_O. Samples of 1 µL volume were cleaned with a Nafion 117 membrane, diluted with 5 µL of H_2_O and 5 µL of methanol plus 1 µL of 1 M H_3_PO_4_ solution (to ensure the complete formation of phosphate adducts in the electrospray spectra) and centrifuged for 1 min at 10 000 **g**. They were examined by ion-mobility, negative-ion MS/MS electrospray ionization on a Waters Synapt G2 mass spectrometer (Waters MS Technologies, Manchester, UK) with sample introduction with Waters thin-wall nanoflow capillaries.

The electrospray ionization capillary voltage was 1.2 kV, the cone voltage was 20–180 V and the source temperature was maintained at 80°C. The T-wave velocity and peak height voltages were 450 m/sec and 40 V, respectively. The T-wave mobility cell contained nitrogen and was operated at a pressure of 0.55 mbar. Fragmentation was performed after mobility separation in the transfer cell with argon as the collision gas. The instrument was externally calibrated with sialylated *N*-glycans released from bovine fetuin. Data acquisition and processing were carried out using the Waters Driftscope (version 2.1) software and MassLynx^TM^ (version 4.0). The scheme devised by Domon and Costello^42^ was used to name the fragment ions and data interpretation followed that described by Harvey *et al*.^43–46^ The diagnostic ions discussed below have all been confirmed by the spectra of known reference compounds. For quantitative measurements, the peak heights of the monoisotopic and isotope ions were summed. The value for each glycan is expressed as a percentage of the total glycans. These data are for relative comparisons only because of the possible differing ion yields from glycans of different types.

### Graphs and statistical analysis

Graphs were generated using GraphPad Prism version 5.04. CC_50_, CC_90_, IC_50_ and IC_90_ values were determined using linear interpolation from graphs on which the percentage inhibition was plotted against increasing drug concentrations. Two-tailed, unpaired *t-*tests were performed using GraphPad Prism version 5.04. The Mann–Whitney rank sum test (Sigma Plot version 12.0) was used to statistically analyse the differences in plaque size measurements. A *P* value <0.05 indicated that the difference between the two datasets was statistically significant.

## Results

### Iminosugars inhibit influenza A virus infection in single-cycle and multicycle assays

#### Cytotoxicity of NB-DNJ and NN-DNJ on MDCK cells

To determine the concentrations of *N*B-DNJ and *N*N-DNJ that can be used to test antiviral activity without affecting cell viability, the CellTiter-Blue™ cell viability assay was used. MDCK cells were treated with increasing concentrations of *N*B-DNJ or *N*N-DNJ or the equivalent amounts of DMSO in serum-free medium for 48 h before the addition of CellTiter-Blue™ reagent, and fluorescence was recorded. Cell viability was not affected at concentrations up to 500 μM *N*B-DNJ and 200 μM *N*N-DNJ. The CC_50_ and CC_90_ values of *N*B-DNJ on MDCK cells could not be specifically determined due to the toxicity of the DMSO diluent so have been described as >1 mM, at which the same concentration of DMSO alone resulted in undetectable cytotoxicity. *N*N-DNJ was more cytotoxic; the CC_50_ and CC_90_ values were 559 ± 112 μM and 859 ± 243 μM (mean ± SD from three independent experiments), respectively.

#### Effects of NB-DNJ, NN-DNJ and NN-DGJ on a single cycle of virus replication

**Figure 1.**
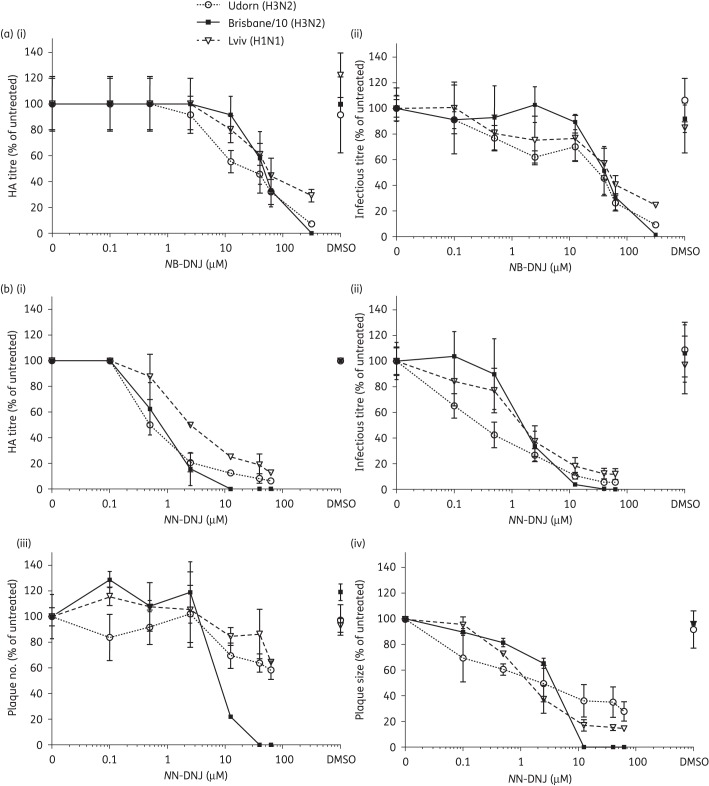
Iminosugar inhibition of single and multicycle replication of different human influenza A viruses. (i) HA titres and (ii) infectious virus titres from single-cycle high-moi assays performed on MDCK cells infected with Udorn, Brisbane/10 and Lviv and treated with (a) *N*B-DNJ and (b) *N*N-DNJ. Mean percentage HA titre or infectious virus titres were calculated as a percentage of HA titre or infectious virus titres, respectively, from untreated cells for each drug treatment condition in an experiment. (b) (iii) Plaque number and (iv) plaque size from plaque reduction assays performed on MDCK cells infected with the three viruses and treated with *N*N-DNJ. Plaques were fixed and stained with toluidine blue. The DMSO control is a DMSO concentration equivalent to the highest concentration of drug tested. In the graphs, the means and standard deviations of three independent experiments are plotted against increasing drug concentration.

The antiviral effects of *N*B-DNJ and *N*N-DNJ on the yield of viruses from cells infected with Udorn, Brisbane/10 and Lviv, at high moi (=10 pfu/cell) and treated with drug after the infection period, were examined. HA titres and infectious virus titres of cell culture supernatants harvested at 20 h pi are shown in Figure 1. The DMSO controls for all three viruses show that DMSO does not affect virus replication in these assays, as shown by both the HA titres and infectious virus titres obtained from DMSO-treated cells compared with untreated cells.

For all three viruses tested, the reduction of virus yield measured by both HA titre and infectivity with increasing concentrations of *N*B-DNJ were similar (Figure 1a i and ii) and IC_50_ and IC_90_ values were calculated for each virus and each drug. The IC_50_ values (Table 1) for HA titre and the release of infectious virus from *N*B-DNJ-treated cells infected with the two H3N2 viruses and the pandemic H1N1 virus were in a similar range, between ∼20–50 μM. At the highest concentration of *N*B-DNJ used in this study, 312.5 μM, infectious virus titres from three independent experiments were reduced to 8%–10% of the untreated control for Udorn, 1%–2% of the control for Brisbane/10 and 23%–25% of the control for Lviv.

**Table 1.** Antiviral effects of *N*B-DNJ, *N*N-DNJ and *N*N-DGJ over single and multiple cycles of replication

	Subtype	Virus	Single-cycle replication assay				Multicycle replication assay			
			HA titre		infectious virus titre		plaque number		plaque size	
			IC_50_ (μM)	IC_90_ (μM)	IC_50_ (μM)	IC_90_ (μM)	IC_50_ (μM)	IC_90_ (μM)	IC_50_ (μM)	IC_90_ (μM)
*N*B-DNJ (μM)	H3N2	Udorn	21.7 ± 15.9	280.0 ± 23.6	34.7 ± 11.2	296.1 ± 16.1	ND	ND	ND	ND
	H3N2	Brisbane/10	43.8 ± 6.5	207.0 ± 95.3	43.6 ± 11.8	250.0 ± 10.4	ND	ND	ND	ND
	H1N1	Lviv	51.3 ± 11.3	>312.5	46.5 ± 12.2	>312.5	ND	ND	ND	ND
*N*N-DNJ (μM)	H3N2	Udorn	0.5 ± 0.0	32.0 ± 14.7	0.4 ± 0.2	16.2 ± 4.7	>62.5	>62.5	6.6 ± 5.5	>62.5
	H3N2	Brisbane/10	1.5 ± 1.0	5.8 ± 1.9	1.73 ± 0.3	10.3 ± 0.3	8.2 ± 2.4	22.0 ± 9.5	4.1 ± 1.2	10.9 ± 0.3
	H1N1	Lviv	2.5 ± 0.0	>62.5	1.9 ± 0.8	>62.5	>62.5	>62.5	1.8 ± 0.3	>62.5
*N*N-DGJ (μM)	H3N2	Udorn	>62.5	>62.5	>62.5	>62.5	>62.5	>62.5	>62.5	>62.5
	H3N2	Brisbane/10	>62.5	>62.5	>62.5	>62.5	>62.5	>62.5	>62.5	>62.5
	H1N1	Lviv	>62.5	>62.5	>62.5	>62.5	>62.5	>62.5	>62.5	>62.5

ND, not determined.

Single-cycle high-moi assays and multicycle plaque reduction assays were performed on MDCK cells infected with Udorn, Brisbane/10 and Lviv. Mean IC_50_ and IC_90_ values were calculated from three independent experiments; ± represents the standard deviation from the mean of three independent experiments.

For each virus tested, as seen with *N*B-DNJ, the reduction in HA titre of virus from the supernatants of drug-treated cells corresponded to the reduction in infectious virus titre from these supernatants with increasing concentrations of *N*N-DNJ. All three viruses had low micromolar IC_50_ values (Table 1). A variation between virus strains was observed in the IC_90_ values. Brisbane/10 had the lowest IC_90_ values when assessed as HA titres and infectious virus titres. IC_90_ values could not be determined for Lviv. At the highest concentration of *N*N-DNJ used, 62.5 μM, the infectious virus titre from three independent experiments was reduced to between ∼5%–7% of the untreated control for Udorn, ∼0.1%–0.4% of the control for Brisbane/10 and ∼11%–14% of the control for Lviv (Figure 1b i and ii). Therefore, Brisbane/10 was the virus that was most susceptible to *N*B-DNJ and *N*N-DNJ. It must be noted that the presence of *N*N-DNJ in the cell culture supernatants did not affect the virus titre measured by plaque or HA assay as described in the Materials and methods section (data not shown).

The antiviral effects of *N*N-DGJ, which is not an α-glucosidase inhibitor but inhibits GSL synthesis, were also determined. IC_50_ values were not obtained at 62.5 μM *N*N-DGJ for the three viruses and no inhibition of virus yield in the single cycle of replication assays was seen. At 62.5 μM *N*N-DGJ for Udorn, Brisbane/10 and Lviv, respectively, the HA titres were 117% ± 19%, 75% ± 15%, 60% ± 10% of the values for untreated cells and the infectious titres were 97 ± 20%, 71 ± 21% and 85% ± 16% of the values seen in untreated cells.

#### Effects of NN-DNJ and NN-DGJ over multiple cycles of replication

The action of *N*N-DNJ on human influenza A virus replication was also examined over multiple cycles of replication using plaque reduction assays. Equivalent concentrations of DMSO did not affect plaque formation in these experiments. For Udorn and Lviv, a <40% reduction in plaque number was observed at the highest concentrations of *N*N-DNJ (Figure 1b iii); hence an IC_50_ was not obtained for plaque number for cells infected with these two viruses. However, in Brisbane/10-infected cells a profound effect of the drug on plaque number was observed. At concentrations of 12.5 μM *N*N-DNJ and higher, an incomplete formation of plaques was observed in the cell monolayers. Toluidine blue staining showed circular areas of lighter stained cells. IC_50_ and IC_90_ values for plaque number were 8.2 ± 2.4 μM and 22.0 ± 9.5 μM, respectively (Table 1). Therefore, *N*N-DNJ inhibited influenza A virus replication in a strain-specific manner over multiple cycles of infection.

The effect of *N*N-DNJ on the size of the virus plaques was also assessed. For all three viruses, plaque size was reduced with increasing concentrations of *N*N-DNJ. Brisbane/10 was the most susceptible to inhibition by *N*N-DNJ (Figure 1b iv) and it was possible to determine IC_50_ and IC_90_ values (Table 1). The IC_50_ values for the median plaque size of all viruses are shown in Table 1, and an IC_90_ value could be determined for the effect of *N*N-DNJ on the release of Brisbane/10 from infected cells. The non-parametric Mann–Whitney rank sum test was used to assess the statistical significance of the reduction in plaque size for *N*N-DNJ-treated cells compared with untreated cells. For Udorn-infected cells treated with a concentration of ≥ 0.1 μM *N*N-DNJ, and for Brisbane/10- and Lviv-infected cells treated with a concentration of ≥0.5 μM *N*N-DNJ, the rank sum analysis showed that the difference in plaque size was statistically significant compared with the untreated control (*P* values in Table S1).

In plaque reduction assays with *N*N-DGJ, neither plaque number nor plaque size was reduced by 50% at any of the concentrations tested; hence IC_50_ values were not obtained for plaque number or plaque size. For Udorn, Brisbane/10 and Lviv, respectively, at 62.5 μM *N*N-DGJ, plaque number was reduced to 74 ± 16%, 60 ± 10% and 73 ± 8% of untreated cells, respectively, and plaque size was reduced to 88 ± 20% and 76 ± 4% for Udorn and Brisbane/10, with no effect on Lviv (104 ± 9% of untreated cells) over three independent experiments.

#### NN-DNJ does not affect virus entry on treatment of cells during infection

In multicycle replication assays, *N*N-DNJ is present on cells throughout the period of infection. *N*N-DNJ inhibits the p7 ion channel of HCV^22,23^ in a genotype-dependent manner,^23^ which does not depend on its glucosidase inhibition but rather is due to the alkyl side chain. Although the HCV p7 and influenza M2 ion channels differ in their structure and function, certain inhibitors such as amantadine have efficacy against both,^47,48^ albeit at different concentrations. Therefore, to examine a potential effect of *N*N-DNJ on the early events of influenza virus replication involving the influenza M2 ion channel, cells were infected with the amantadine-susceptible strain Udorn in the presence of amantadine, as a control, or *N*N-DNJ (Figure S1a), or were infected with the three different influenza strains in the presence of *N*N-DNJ (Figure S1b) with drug also present after infection. Infected cells were quantified by counting the number of influenza NP-positive cells at 6 h pi. Amantadine treatment resulted in a significant reduction in the number of NP-positive cells to ∼14% of the untreated control. However, in these experiments, *N*N-DNJ had at most only a minor impact on the ability of virus to initiate infection.

### NN-DNJ reduces release of virus particles from cells

**Figure 2.**
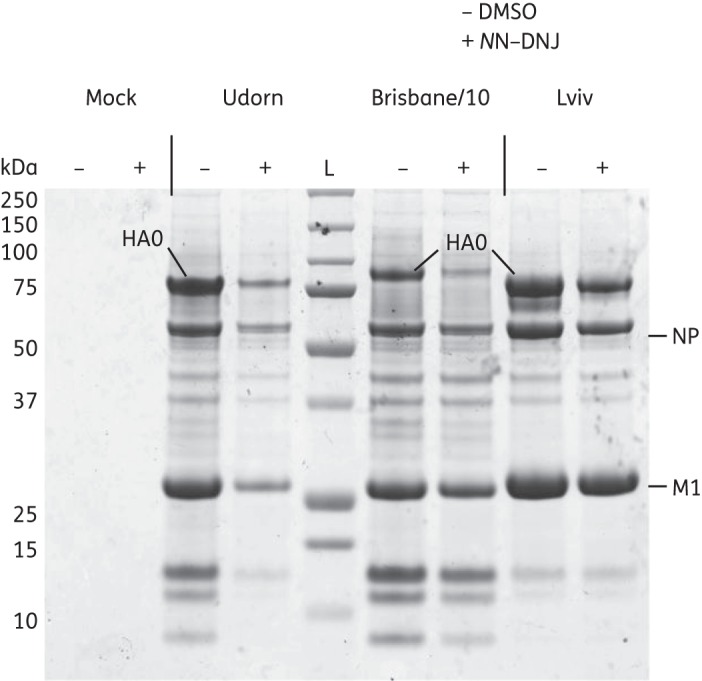
SDS-PAGE gel showing virus purified from cell culture supernatants of *N*N-DNJ-treated cells. Single-cycle high-moi assays were performed on MDCK cells infected at an moi of 10 pfu/cell with Udorn, Brisbane/10 and Lviv and treated with 62.5 μM *N*N-DNJ or an equivalent DMSO control. At 19 h pi for Brisbane/10 and Lviv and 11 h pi for Udorn, cell culture supernatants were harvested. Virus was purified from supernatants by ultracentrifugation through a 30% sucrose cushion. Proteins from pellets were separated by SDS-PAGE on a 10% polyacrylamide gel under reducing conditions and protein bands (HA0, NP and Matrix protein M1) were visualized by Coomassie blue staining.

*N*N-DNJ reduced the release of infectious virus from the cells and the HA titres corresponded to this reduction in infectivity (Figure 1b). In order to determine whether *N*N-DNJ affects the total release of virus-like particles that might not be either infectious or able to bind to red blood cells, single-cycle high-moi virus yield assays were performed, treating the cells after infection with 62.5 μM *N*N-DNJ or the equivalent DMSO control in the supernatant, and the production of virus particles was assessed. Virus particles from clarified supernatants were pelleted by ultracentrifugation through a 30% sucrose cushion. Equal volumes from each sample were taken and proteins from purified virus were separated by SDS-PAGE under reducing conditions (Figure 2). It is not possible to estimate the levels of virus protein between treatments due to possible handling losses during virus purification. However, the ratio of stained uncleaved HA, HA0, to NP can be estimated within a sample. This was done for two independent experiments. For Lviv there was no discernible difference between *N*N-DNJ- and control DMSO-treated cells, but for both Udorn and Brisbane/10 there was a recognizable drop in the HA content of the virion, manifested by a reduction in the ratio of Coomassie-blue-stained HA to NP by between one-third and two-thirds for both Udorn and Brisbane/10.

To quantify further the reduction in the number of virus particles purified from *N*N-DNJ-treated cells, qRT–PCR was performed on RNA extracted from virus pellets that had been obtained from the same amount of cell culture supernatant for each sample. The levels of influenza Segment 7 (encoding M) from purified virus from *N*N-DNJ-treated cells over three independent experiments were 4%–13%, 0.5%–3%, and 15%–40%, for Udorn, Brisbane/10 and Lviv in drug-treated cells, respectively, compared with the control DMSO-treated cells. Fewer virus particles are released from drug-treated cells and it appears that *N*N-DNJ treatment reduces the titre of infectious virus released (Figure 1 and Table 1) by reducing the total number of virions released (shown by SDS-PAGE and qRT–PCR). For Udorn, Brisbane/10 and Lviv, respectively, HA titres from *N*N-DNJ-treated cells were 6%–8%, 0% and 13%–25% of the control and infectious titres were 6%–8%, 0.2%–0.8% and 12%–20% of the control. With the overlap of these values for each virus as assessed by SDS-PAGE or RT–PCR, there is no evidence to suggest the presence of a sizeable proportion of virus that is released from the cell but is unable to initiate further replication.

### Analysis of virus glycoproteins synthesized in NN-DNJ-treated cells shows alteration in glycan processing

To examine the effects of *N*N-DNJ on the glycoprotein processing of influenza A virus glycoproteins, MDCK cells infected with Udorn, Brisbane/10 and Lviv and treated with *N*N-DNJ or the equivalent DMSO control were metabolically labelled with [^35^S]methionine/cysteine at 6 h pi for 40 min to allow time for synthesis and post-translational modification. Infected cell lysates were separated by SDS-PAGE under reducing conditions (Figure S2). For all three viruses, a reduction in electrophoretic mobility of HA0 synthesized in *N*N-DNJ-treated cells was seen when compared with DMSO-treated cells, consistent with α-glucosidase inhibition by *N*N-DNJ and a corresponding effect on the mobility of HA0. Importantly, there was no marked difference in the amounts of either HA0 or NP synthesized over this 40 min period between drug-treated samples and the DMSO control.

**Figure 3.**
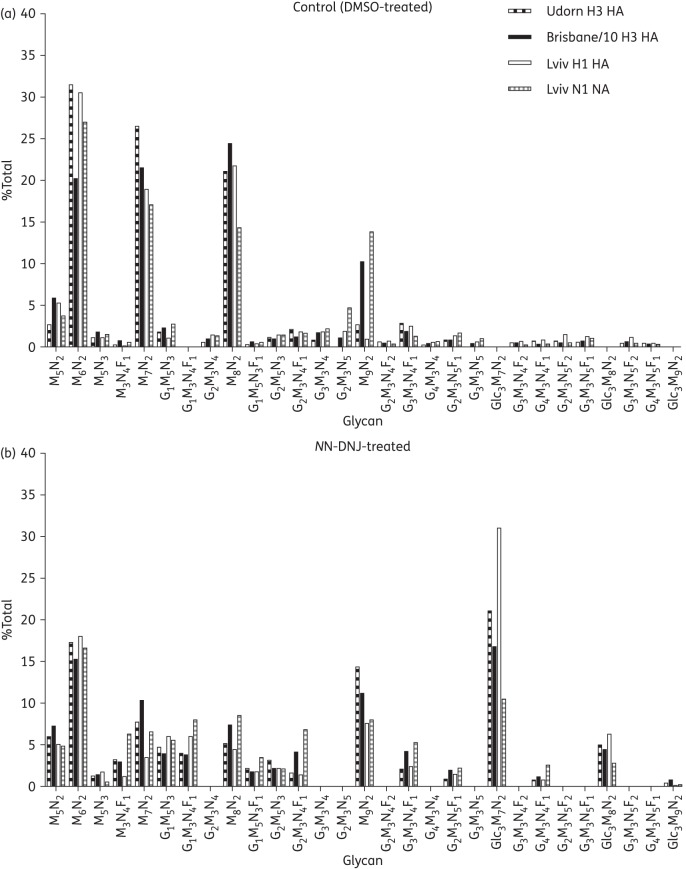
MS analysis of the glycan composition of influenza glycoproteins from virus purified from *N*N-DNJ-treated cells. MDCK cells were infected at an moi of 10 pfu/cell with Udorn, Brisbane/10 and Lviv and treated with 62.5 μM *N*N-DNJ (b) or the equivalent DMSO control (a). Virus from cell culture supernatants was purified by ultracentrifugation, proteins from pellets were separated by SDS-PAGE, glycoprotein bands were cut and digested glycans were analysed by MS as described in the Materials and methods section. Glycans (M = mannose, G = galactose, Glc = glucose, N = *N*-acetyl glucosamine, F = fucose) from Udorn HA, Brisbane/10 HA and Lviv HA and NA are expressed as a percentage of the total glycans.

MS analysis was used to compare the glycan composition of the influenza glycoproteins from virus purified from *N*N-DNJ-treated and control (DMSO-treated) cells (Figure 3 and Table S2). Glycans from the untreated cells were predominantly high-mannose oligosaccharides with differences in the glycan composition of HA in each of the strains: Brisbane HA had a higher proportion of Man_9_GlcNAc_2_ (M_9_N_2_) (∼10%) in comparison with Lviv and Udorn (1%–3%) but a lower percentage of M_6_N_2_. Iminosugar treatment increased the proportion of tri-glucosylated glycans (Glc_3_H_7_N_2_, Glc_3_H_8_N_2_ and Glc_3_H_9_N_2_) from undetectable in the control DMSO-treated samples to 26.4% (Udorn), 21.8% (Brisbane) and 37.3% (Lviv) of total glycans, respectively, in HA from virus grown in the presence of *N*N-DNJ, producing a substantial change in the glycan profiles consistent with an inhibition of ER α-glucosidases

### Influenza H3 HA synthesized in NN-DNJ-treated cells shows reduced surface expression

**Figure 4.**
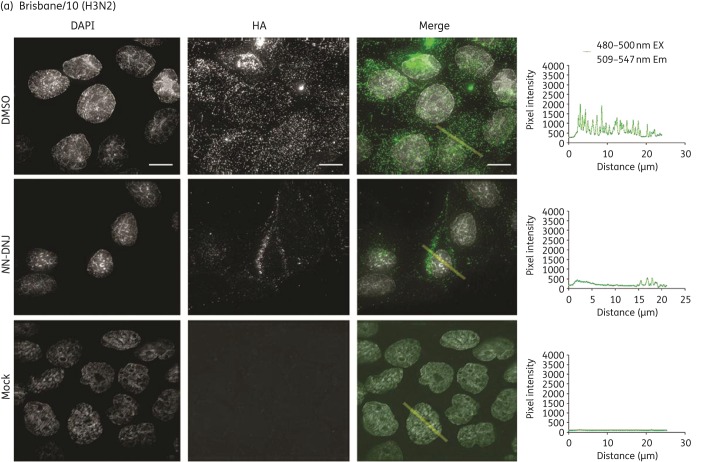

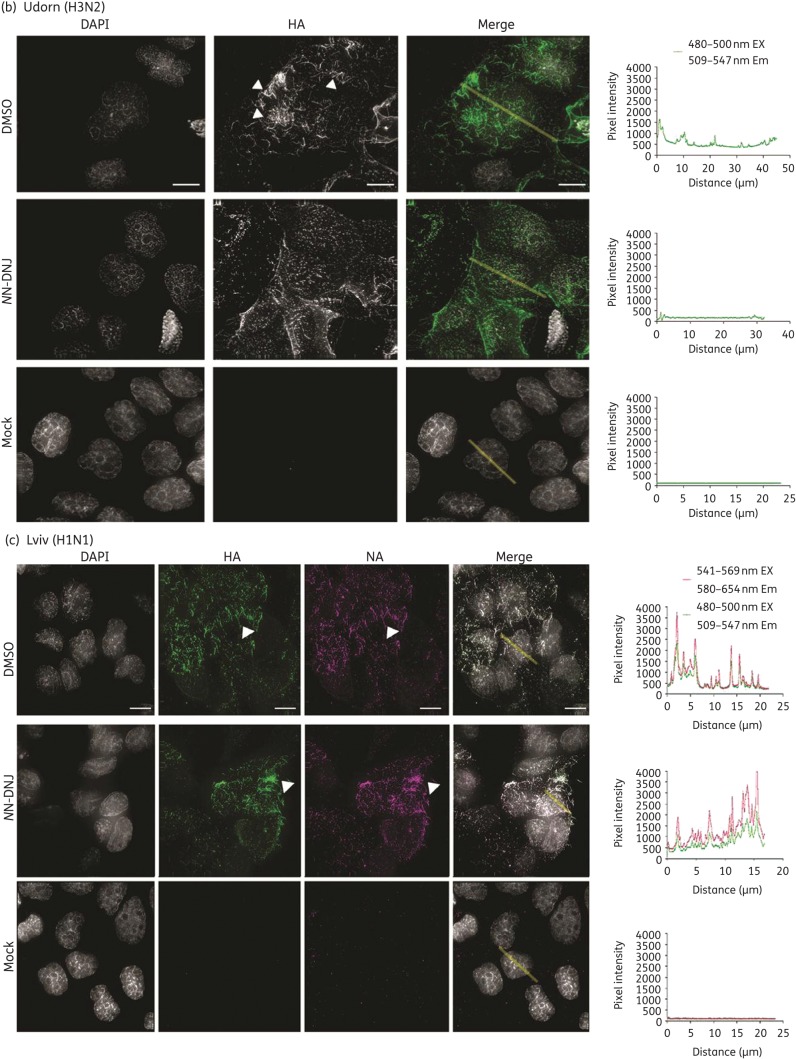
Surface expression of HA or NA in cells infected with Udorn, Brisbane/10 or Lviv treated with or without *N*N-DNJ. MDCK cells were infected at an moi of 5 pfu/cell with (a) Brisbane/10, (b) Udorn and (c) Lviv and treated with 62.5 μM *N*N-DNJ or the equivalent DMSO control. At 8 h pi, the cells were fixed and immunostained for HA or NA. Images were acquired on a DeltaVision RT microscope using a ×100 objective (numerical aperture = 1.4) obtaining Z-sections of 0.2 μm to a depth of 7 μm. Images were deconvolved and 2D maximal intensity projections were generated using Fiji software. In the Udorn and Brisbane/10 merge images, HA is shown in green. In the Lviv single panels and Merge images, HA is shown in green and NA in magenta. Bars, 10 μm. A line (shown in yellow) was drawn across a cell where indicated and plots of pixel intensity versus distance (μm) for the line are shown next to the relevant image; the quantification of 30 such cells per treatment condition is shown in Figure S3. Arrow heads indicate filamentous virions.

Light microscopy was used to visualize the surface expression of influenza glycoproteins from immunostained *N*N-DNJ-treated cells. Figure 4(a) shows the immunostaining of surface HA from Brisbane/10-infected MDCK cells treated with 62.5 μM *N*N-DNJ or the equivalent DMSO control. HA is seen to be present within foci in the control cells and a visible reduction of HA incorporation into foci was observed in *N*N-DNJ-treated cells. Fluorescence on the cell surface was quantified from images by measuring the overall fluorescent intensity from the cells (Figure S3a, with an illustrated example shown in Figure 4) and then plotting it as the number of fluorescent pixels per unit area for each cell versus the cell treatment condition. A two-tailed unpaired *t*-test showed that there was a statistically significant reduction in the number of fluorescent pixels per unit area for cells treated with *N*N-DNJ compared with the DMSO-treated control cells (*P* < 0.0001). Light microscopy of immunostained surface HA on Udorn-infected MDCK cells in Figure 4(b) shows filamentous virions (indicated by arrow heads). As seen with Brisbane/10, a visible reduction in HA incorporation into foci was observed for *N*N-DNJ-treated cells compared with DMSO-treated cells, and a reduction in viral filaments incorporating HA was observed. A quantification of overall fluorescence of Udorn HA on the surface of DMSO-treated cells (Figure S3b) showed that the number of fluorescent pixels per unit area from different cells was more variable for Udorn than for Brisbane/10 (Figure S3a) and this difference is likely to be due to the more varied morphology of the budding virus structures observed. The Mann–Whitney rank sum test showed a statistically significant reduction in the number of fluorescent pixels per unit area for cells treated with *N*N-DNJ compared with the DMSO-treated control cells (*P* < 0.0001).Therefore, a reduction in the surface expression of HA on Udorn was observed from *N*N-DNJ-treated cells compared with the control.

The surface expression of both HA and NA for Lviv was visualized by immunostaining and light microscopy (Figure 4c). Co-localization of HA and NA was observed, as shown in the merged figure. A quantification of the overall fluorescence intensity of HA and NA on the surface of cells in Figure S3(c) (i and ii) showed no significant difference (by Mann–Whitney rank sum test) between drug-treated and DMSO-treated cells.

These results demonstrate a reduction in the expression of surface HA on *N*N-DNJ-treated cells infected with Udorn and Brisbane/10 when compared with DMSO-treated cells, but *N*N-DNJ had no discernible effect on the surface expression of HA or NA for Lviv.

### Influenza NA synthesized in NN-DNJ-treated cells shows reduced sialidase activity

To investigate the antiviral effects of an *N*N-DNJ-mediated alteration of glycan processing on the functionality of NA in *N*N-DNJ-treated cells, the enzymatic activity of NA synthesized in *N*N-DNJ-treated cells was compared with that in DMSO-treated cells. MDCK cells were infected with Udorn, Brisbane/10 or Lviv at an moi of 5 pfu per cell and treated with 62.5 μM *N*N-DNJ or the equivalent DMSO control. The sialidase activity of NA synthesized in cells was examined by a MuNANA assay of post-nuclear supernatants harvested at several time points pi. A reduction in sialidase activity was seen from NA synthesized in *N*N-DNJ-treated cells for all three viruses when compared with the DMSO control. For Udorn, Brisbane/10 and Lviv, the reduction in sialidase activity of NA from *N*N-DNJ-treated cells was ∼35%–45%, ∼30%–40% and ∼45%–60%, respectively, compared with the DMSO control over three independent experiments.

Therefore, although a reduction in the sialidase activity of NA synthesized from cells treated with the inhibitory concentration of 62.5 μM *N*N-DNJ was observed, the reduction did not follow the strain-specific pattern of an *N*N-DNJ-mediated inhibition of virus release.

### Antiviral effect of NN-DNJ on virus replication is not complemented by the addition of an exogenous sialidase

To address whether the reduction in sialidase activity of NA synthesized in *N*N-DNJ-treated cells played a role in the reduction in the release of virus particles from the cell surface, the ability of exogenous NA added to the medium to complement sialidase activity was examined.

**Figure 5.**
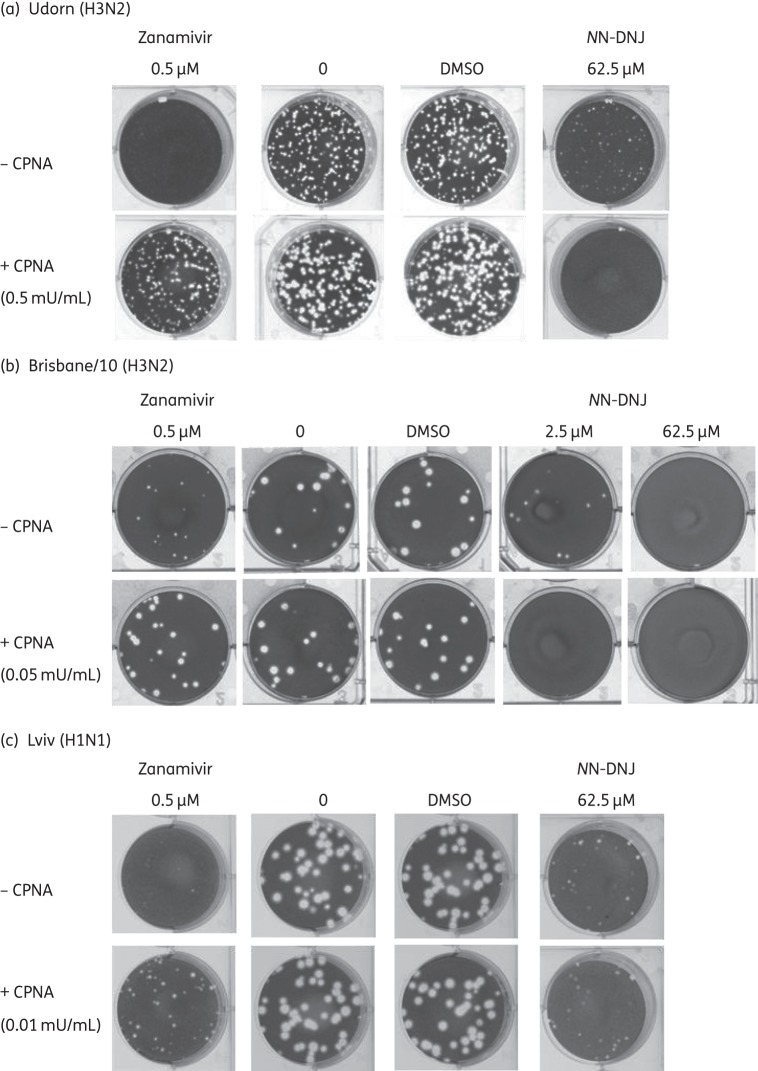
Addition of exogenous CPNA in plaque reduction assays does not restore the plaque size in cells treated with *N*N-DNJ. MDCK cells were infected with (a) Udorn, (b) Brisbane/10 and (c) Lviv and plaque reduction assays were performed. Concentrations of reagents in the plaque assay overlay are indicated in the figure. Cells were fixed and stained with toluidine blue at 30 h pi for Udorn, 48 h pi for Brisbane/10 and 40 h pi for Lviv.

Figure 5 shows representative plaque reduction assays. In Udorn-, Brisbane/10- and Lviv-infected cells, the addition of 0.5 mU/mL CPNA, 0.05 mU/mL CPNA and 0.01 mU/mL CPNA, respectively, restored plaque formation in the presence of zanamivir, showing that exogenous NA complemented the zanamivir-mediated inhibition of sialidase activity. For Udorn, the addition of CPNA to untreated and DMSO-treated cells resulted in a discernible increase in plaque size; for the other two viruses, however, there was no difference in plaque size between the untreated control cells or DMSO-treated control cells with or without CPNA as assessed by the Mann–Whitney rank sum test (data not shown). For all three viruses, the reduction in plaque size with inhibitory concentrations of *N*N-DNJ was not enhanced by the addition of CPNA. Interestingly, for both Udorn and Brisbane/10 but not for Lviv, plaque formation at these *N*N-DNJ concentration(s) was inhibited by CPNA. For Lviv, the difference in plaque size between cells treated with 62.5 μM *N*N-DNJ with or without CPNA was not statistically significant by Mann–Whitney rank sum test (data not shown).

These results show that the addition of exogenous NA does not restore plaque size in *N*N-DNJ-treated cells. Therefore, the reduction in sialidase activity observed for all three viruses seems not to be a major contributor to the *N*N-DNJ inhibition of virus replication in cultured cells.

### HA is the major determinant of virus susceptibility to NN-DNJ

The distinctive pattern of inhibition of the three viruses by *N*N-DNJ was further examined using reassortant viruses to determine whether the inhibitory pattern correlated with the subtype of the glycoproteins. First, the effect of *N*N-DNJ on two high-growth reassortant vaccine viruses, X-181 (H1N1) and X-171b (H3N2), reassortant viruses that differ only in their HA and NA genes, was tested. X-181 contains the HA and NA of the pandemic H1N1 A/California/7/2009 (HA has 99% sequence identity and NA has 99.6% sequence identity with Lviv, with no differences in potential glycosylation sites), and X-171b has the HA and NA of Brisbane/10 (H3N2). Both viruses contain the same six internal genomic segments derived from PR8.

**Figure 6.**
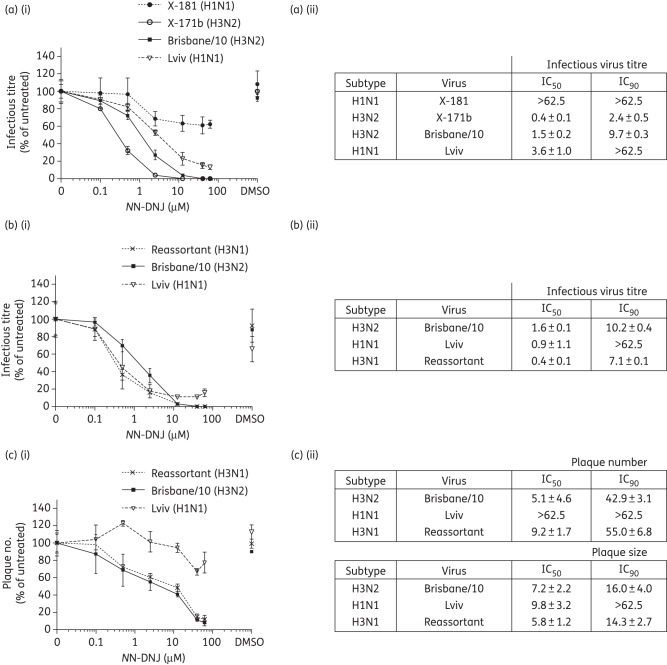
Susceptibility of reassortant viruses to *N*N-DNJ compared with wild-type viruses. Infectious virus titres from single-cycle high-moi assays performed on MDCK cells infected with (a) high-growth vaccine reassortants X-181 and X-171b and wild-type viruses Brisbane/10 and Lviv or (b) H3N1 reassortant and wild-type viruses Brisbane/10 and Lviv and then treated with *N*N-DNJ. (c) Plaque reduction assays were performed on MDCK cells infected with the H3N1 reassortant and wild-type viruses Brisbane/10 and Lviv and then treated with *N*N-DNJ. Plaques were fixed and immunostained for NP. In the graphs on the left, means and standard deviations of infectious virus titre of three (a) and two (b and c) independent experiments are plotted against increasing drug concentration. On the right are summary tables of mean IC_50_ and IC_90_ values from independent experiments described in (a), (b) and (c), respectively; ± represents the standard deviations (a) or ranges (b and c) from the mean.

Single-cycle high-moi assays were performed with X-181, X-171b, Brisbane/10 and Lviv. Figure 6(a) shows the release of infectious virus from MDCK cells infected with the four viruses and treated with increasing concentrations of *N*N-DNJ. X-171b, with its HA and NA genes derived from Brisbane/10, was the most susceptible to inhibition by *N*N-DNJ and X-181 (H1N1) was the least susceptible to *N*N-DNJ (Figure 6a), with each virus showing a different profile. Their IC_50_ and IC_90_ values are shown in Figure 6(a) (ii). From these results, the effects of *N*N-DNJ on virus production are determined by the source of the HA and NA genes. Although viruses containing the same subtype of HA and NA but different internal segments (the reassortant versus its respective wild-type) differed in susceptibility, the reassortant X-171b and X181 viruses, which contain identical internal genes but have HA and NA genes from different subtypes, showed dramatic differences in susceptibility when compared, reflecting the susceptibilities seen in the wild-type viruses.

A reassortant H3N1 virus generated between Brisbane/10 (H3N2) and Lviv (H1N1) that contained seven segments from Brisbane/10 and Segment 6 (encoding NA) from Lviv was used to determine which segment predominantly determines the *N*N-DNJ susceptibility profile of the virus. The effect of *N*N-DNJ on this reassortant H3N1 virus was compared with the susceptibility of the parent viruses Brisbane/10 and Lviv to *N*N-DNJ. Single-cycle replication assays were performed with all three viruses (Figure 6b). The reassortant H3N1 was the most susceptible to *N*N-DNJ of all three viruses tested in this experiment. IC_50_ values were obtained for all three viruses (Figure 6b ii). The mean IC_50_ values for the three viruses were within 4-fold of each other; however, the IC_90_ values for both the reassortant H3N1 and Brisbane/10 were comparable and an IC_90_ value was again not obtained for Lviv. The effects of *N*N-DNJ on the multicycle replication of the reassortant H3N1 virus were determined by plaque reduction assay (Figure 6c). A similar trend for reduction in plaque number was observed for Brisbane/10 and the reassortant H3N1 (Figure 6c). A small reduction in plaque number was observed for Lviv at the higher concentrations of *N*N-DNJ (as shown in Figure 1b iii). This pattern is reflected in the IC_50_ and IC_90_ values summarized in the table in Figure 6(c) (ii). These results highlight the similarity in susceptibility to *N*N-DNJ of Brisbane/10 and the reassortant H3N1. While all three viruses had similar IC_50_ values for plaque size, IC_90_ values for plaque size obtained for the reassortant H3N1 and Brisbane/10 coincide (Figure 6c ii).

Therefore, the reassortant H3N1 virus was extremely susceptible to *N*N-DNJ, as is Brisbane/10, over multiple cycles of replication. These results indicate that the HA gene most likely determines *N*N-DNJ susceptibility, as the NA of Lviv was not sufficient to confer resistance to the H3N1 reassortant.

## Discussion

We show that two iminosugars that are known α-glucosidase inhibitors, *N*B-DNJ and *N*N-DNJ, exhibit antiviral effects on three human influenza A viruses: two A(H3N2) viruses Udorn and Brisbane/10, and the A(H1N1)pdm09 Lviv. An iminosugar *N*N-DGJ, which is not an inhibitor of α-glucosidases, showed much weaker inhibition. When the effects of the two α-glucosidase inhibitors were assessed over a single cycle of virus replication, *N*N-DNJ was more effective, with an IC_50_ over 10-fold lower than that observed for *N*B-DNJ. To examine the more potent inhibitor, *N*N-DNJ, in more detail, the inhibition of virus replication was examined over multiple cycles of replication by plaque formation, assessing both plaque number and the size of the virus plaques. Brisbane/10 was strongly inhibited by *N*N-DNJ in terms of plaque number, although Udorn and Lviv showed only a small reduction in absolute plaque number with *N*N-DNJ treatment but a significant reduction in plaque size. Several previous studies have reported that α-glucosidase inhibition did not affect the release of infectious influenza virus,^31,33,34,50^ but in these studies the inhibitors were different and the virus strains used were laboratory strains from the 1930s. Some strain-specific effects of α-glucosidase inhibition have been observed in a reassortant H1N8 virus^35^ and in an H7N1 subtype virus with an inhibitor of α-glucosidase II, BC.^33^ Our studies used more recently circulating viruses and more recently developed α-glucosidase inhibitors.

*N*N-DNJ, having a longer alkyl chain than *N*B-DNJ, showed increased cytotoxicity as well as increased antiviral effects at non-cytotoxic concentrations. This increased toxicity with increasing chain length is a general trend shown by iminosugar derivatives; such effects have been reported by others against different viruses grown in various cell lines,^17,18,51^ and correlate with increased cellular uptake and retention.^18,52,53^ The stronger antiviral effect of DNJ derivatives with long alkyl chains are possibly due to side-chain effects.^22,51,54^

Consistent with its action as an inhibitor of α-glucosidases in the ER, *N*N-DNJ treatment caused an increase in the amount of tri-glucosylated structures on influenza glycoproteins, as assessed by the quantitative MS analysis of glycan composition. The detection of these structures on secreted virus suggests that some HAs and NAs were not misfolded sufficiently to lead to retention in the ER (where this results in accumulation or ER-associated degradation) and may reflect regional or partial misfolding of these glycoproteins. Such effects have been seen previously with, for instance, tyrosinase, which still traffics to melanosomes following α-glucosidase inhibition-induced misfolding^55^ or with *N*B-DNJ-induced localized glycoprotein misfolding confined to the V1/V2 loops of HIV gp120.^13^ While Lviv HA grown in the presence of *N*N-DNJ had the highest proportion of hyperglucosylated structures, many other factors are likely to influence susceptibility to iminosugars and this is not evidence that the Lviv strain is more susceptible to α-glucosidase inhibition. In fact, our *in vitro* data suggest that Lviv is the least susceptible to iminosugars of the three strains tested. The Brisbane/10 HA contained a higher proportion of high-mannose glycans, in comparison to Udorn and Lviv HA, which may reflect the reduced accessibility of mannose-trimming enzymes. No bi-glucosylated structures were detected, consistent with the strong inhibition of α-glucosidase I by *N*N-DNJ. The distributions of glycoforms were similar to those quantified on MDCK-grown A/Memphis/1/71 (H3N2),^56^ such as the relatively high proportion of high-mannose glycans and the presence of α-galactose and bisected structures with a core fucose.

The effects of an *N*N-DNJ-mediated alteration of glycan processing of HA and NA on the influenza A life cycle were examined. Protein synthesis in drug-treated cells was not markedly affected, as assessed by the metabolic labelling of virus polypeptides (data not shown). This observation is in line with those in other studies, which have shown that treatment with DNJ derivatives does not affect the protein synthesis of BVDV^19,51^ or HCV^17^ and the treatment of MDCK cells with the iminosugars CST, *N*M-DNJ, homonojirimycin and *N*-methyl-homonojirimycin does not affect the synthesis of influenza A proteins.^31,34,57^ Nevertheless, a reduction in HA expression of Udorn and Brisbane/10 on the surface of infected cells was observed by immunofluorescence microscopy on treatment with *N*N-DNJ but this was not seen in cells infected with Lviv. The surface expression of the NA of Lviv from *N*N-DNJ-treated cells was also not reduced when compared with control cells (immunostaining of NA from Udorn- and Brisbane/10-infected cells could not be performed due to a lack of suitable antibodies). Therefore, the *N*N-DNJ-mediated reduction in HA expression on the cell surface differed between virus strains. Previous studies have shown that α-glucosidase inhibition by DNJ or CST did not reduce the cell surface expression of HA^28,35,50^ but in those studies prototype viruses first isolated in the 1930s and propagated extensively since then were examined and different inhibitors were used.

Both HA and NA bind to calnexin and calreticulin for proper folding^24–29^ and misfolded proteins may not be trafficked correctly in *N*N-DNJ-treated cells. Our observed reduction in the sialidase activity of NA synthesized in *N*N-DNJ-treated cells could be due to a reduction in mature NA synthesized in cells for the H3N2 viruses or due to a reduction in the enzymatic activity of a misfolded protein, but we are currently unable to differentiate between these possibilities. Nevertheless, a reduced activity of sialidase could not be complemented by the addition of an exogenous bacterial NA, unlike the zanamivir-induced inhibition of the release of virus.^36,58^ These results suggest that the *N*N-DNJ inhibited virus replication at the assembly and/or budding stage, rather than at the release of virus particles from infected cells.

The two α-glucosidase inhibitors *N*B-DNJ and *N*N-DNJ showed strain-specific antiviral effects whereas the non α-glucosidase inhibitor *N*N-DGJ did not. Reassortant viruses that have a PR8 background but carry the HA and NA genes of circulating viruses show that the strain-specific pattern of inhibition correlated with the origin of the virus glycoproteins. We observed that the reduction in sialidase activity of NA synthesized in *N*N-DNJ-treated cells did not follow the strain-specific pattern of the *N*N-DNJ-mediated inhibition of release of virus, and that the reassortant H3N1 virus showed similar susceptibility to Brisbane/10 (H3N2) in IC_90_ values on *N*N-DNJ treatment, indicating that drug susceptibility is most likely determined by the HA, and that the NA of Lviv was not able to confer relative resistance on this reassortant; this indicates that the NA is not the main determinant of *N*N-DNJ inhibition. However, since the reciprocal reassortant H1N2 virus has not been made and tested for drug susceptibility, it cannot be excluded that such a virus may show an effect similar to the H3N1 virus on drug treatment.

We hypothesize that the differential susceptibility of viral strains to *N*N-DNJ treatment may reflect a differential sensitivity to misfolding or to the consequences of misfolding in any region where it might occur. For example, any effects of regional misfolding around the receptor-binding site could alter the specific ratio of HA and NA enzymatic activity to affect the infectivity of strains differentially. Another potential mechanism to explain differential strain susceptibility is a variation in assembly of the HA trimers between strains, with some accommodating misfolding to a greater or lesser extent. Trimerization is a sequence-specific event^55^ so differences in the efficiency of oligomerization of the HA subunits for individual strains may enhance the α-glucosidase sensitivity such that we can detect differences in the overall antiviral effect. It is notable that we have observed some differences between the viruses in the relative incorporation efficiency of the HA into virus particles on treatment with *N*N-DNJ, which might be due to misfolding and to a failure to reach the surface of the cell for assembly and budding. This agrees with the observations presented and discussed above on the cell surface expression seen on immunofluorescence microscopy.

It is noteworthy that the glycoproteins of different strains of influenza virus can differ in the number of N-linked glycosylation sites and may also differ in their requirement for glucose-trimming for maturation. Of the strains used in this study, the recent A(H3N2) virus Brisbane/10, which was the most susceptible to *N*N-DNJ inhibition, has acquired five more potential N-linked glycosylation sites in the HA compared with the older Udorn, as previously noted.^59^ These extra five glycosylation sites lie in the head region (reviewed in Skehel and Wiley^3^). Notably, the pandemic A(H1N1) virus does not have any glycosylation sites in the head region. The NA glycoproteins of the three viruses have a similar number of glycosylation motifs: Brisbane/10 and Lviv having eight and Udorn seven potential N-linked glycosylation sites.

We have yet to explain the inhibition in cell biological terms. For example, evidence implies that influenza viruses bud from lipid rafts (reviewed in Rossman and Lamb^60^). Budding defects may occur due to a reduction in the targeting of viral glycoproteins to lipid rafts due to an alteration in the lipids. DNJ derivatives with a minimum alkyl chain length of at least three carbons can inhibit CerGlcT, inhibiting GSL synthesis^11^ (as do DGJ derivatives), thus potentially affecting raft formation. Another level at which these iminosugars may be affecting influenza replication may be on virus entry due to a reduction in the number of virus receptors, sialic acids, expressed on glycoconjugates on the cell surface by an inhibition of GSL synthesis (DNJ and DGJ derivatives) and α-glucosidase inhibition (DNJ derivatives). Future work will include examining relative viral protein turnover levels in cells and characterizing the effects of iminosugars on glycoprotein folding, trimerization and function, in addition to the effects on virus entry and assembly.

If the safety and efficacy of iminosugars as antivirals for influenza in humans were to be established, these drugs might be administered in combination with drugs targeting viral proteins to patients suffering from severe illness, and alone to those infected with multiple drug-resistant strains or those who are unable to take other available anti-influenza drugs due to underlying complications.

## Funding

This work was funded by the Medical Research Council UK through programmes U117585868 and U117581334. This work was further supported by the Oxford Glycobiology Endowment (J. L. M. and N. Z.) and a ‘Blue Skies’ grant from United Therapeutics Corporation. N. Z. is a Fellow of Merton College, Oxford.

## Transparency declarations

None to declare.

## Supplementary data

Primer sequences, Table S1, Table S2 and Figures S1 to S3 are available as Supplementary data at *JAC* Online (http://jac.oxfordjournals.org/).

Supplementary Data
