# Supplementary material for: Strain-specific antiviral activity of iminosugars against human influenza A viruses
Source: J Antimicrob Chemother. 2014 Sep 15;70(1):136–52. doi: 10.1093/jac/dku349 (PMC4267503; doi:10.1093/jac/dku349)
Supplement: Supplementary Data [file supp_70_1_136__index.html]

Strain-specific antiviral activity of iminosugars against human influenza A viruses — Strain-specific antiviral activity of iminosugars against human influenza A viruses — Supplementary Data 

# Strain-specific antiviral activity of iminosugars against human influenza A viruses

## Supplementary Data

Supplementary Data

**Files in this Data Supplement:**

- Supplementary Data - Docx file
