# Supplementary material for: Strain-specific antiviral activity of iminosugars against human influenza A viruses
Source: J Antimicrob Chemother. 2014 Sep 15;70(1):136–52. doi: 10.1093/jac/dku349 (PMC4267503; doi:10.1093/jac/dku349)
Supplement: Supplementary Data [file supp_dku349_dku349supp.docx]

**Supplementary data**

**

**

Figure S1. *N*N-DNJ does not affect the infection of cells with three viruses, assessed by quantification of NP positive cells. **(a)** MDCK cells were infected at an moi of 0.1 pfu/cell with Udorn in presence of increasing concentrations of *N*N-DNJ or amantadine, and **(b)** Udorn, Brisbane/10 or Lviv, in the presence of increasing concentrations of *N*N-DNJ. The drug was present during infection and for 6 hours p.i. Cells were fixed and immunostained for influenza NP. NP positive cells were quantified. Mean and standard deviations of percentage NP positive cells of three independent experiments is plotted versus increasing *N*N-DNJ concentration. *P* values are indicated.


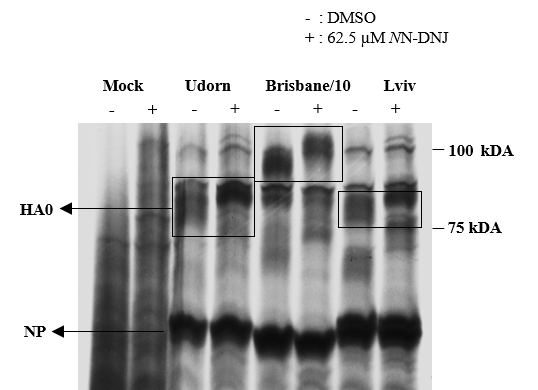


Figure S2. Metabolic labelling of proteins synthesised in *N*N-DNJ-treated MDCK cells infected with Udorn, Brisbane/10 and Lviv. MDCK cells were infected with Udorn, Brisbane/10 or Lviv at an moi of 10 pfu/cell and treated after infection with 62.5 µM *N*N-DNJ or the equivalent DMSO control in serum-free medium. At 6 hours p.i., cells were metabolically labelled with ^35^S Met-Cys in Met-Cys-free medium with *N*N-DNJ or the DMSO control. After a 40 min pulse, cells were lysed and the proteins were separated by SDS-PAGE on a 10% polyacrylamide gel under reducing conditions. Labelled proteins were detected by autoradiography. Autoradiograph of the gel with the HA0 and NP of all three viruses indicated.





Figure S3. Quantification of cell surface expression of proteins from *N*N-DNJ-treated cells. Maximal 2D intensity projections from Z-stacks acquired on Deltavision RT microscope and deconvolved, were generated using Fiji software. Each cell was outlined, selected and analysed for fluorescence intensity. The overall fluorescence intensity for each cell is plotted as the number of fluorescence pixels versus µm^2^ for cells treated with 62.5 µm *N*N-DNJ (filled squares) or DMSO-treated control cells (filled circles). Fifteen cells from each sample were quantified from a representative experiment. The line represents the mean value. Light transmission and exposure settings for imaging were fixed for each virus. *P* values are indicated.

|  | ***P* value** | | |
| --- | --- | --- | --- |
| ***N*N-DNJ (µM)** | **Udorn** | **Brisbane/10** | **Lviv** |
| 0.1 | 0.043— < 0.001 | 0.070—0.131 | 0.301—0.184 |
| 0.5 | < 0.001 | 0.006—0.014 | 0.001—0.005 |
| 2.5 | < 0.001 | < 0.001 | < 0.001 |
| 12.5 | < 0.001 | < 0.001 | < 0.001 |
| 40 | < 0.001 | < 0.001 | < 0.001 |
| 62.5 | < 0.001 | < 0.001 | < 0.001 |
| DMSO control | 0.935—0.661 | 0.309—0.789 | 0.917—0.583 |

**Table S1. Mann-Whitney Rank sum analysis of difference in plaque size from *N*N-DNJ-treated cells compared with untreated cells.** Plaque reduction assays were performed on MDCK cells infected with Udorn, Brisbane/10 and Lviv. 30 plaques were measured (to the nearest 0.1 mm) for each cell treatment in an experiment. The plaque size difference from *N*N-DNJ treated cells was compared with that from untreated cells using the Mann-Whitney Rank sum test and *P* value ranges over three independent experiments are shown.

| ***m/z*^a^**  **[M+H_2_PO_4_]^-^** | **Composition^b^** | **DMSO-treated (Control)** | | | | ***N*N-DNJ-treated** | | | | **Structure or proposed structure^c,d^** |
| --- | --- | --- | --- | --- | --- | --- | --- | --- | --- | --- |
|  |  | **Udorn HA** | **Brisbane/10 HA** | **Lviv HA** | **Lviv NA** | **Udorn HA** | **Brisbane/10 HA** | **Lviv HA** | **Lviv NA** |  |
| 1331 | M_5_N_2_ | *2.6* | *5.9* | *5.3* | *3.7* | 6.0 | 7.2 | 5.0 | 4.8 | 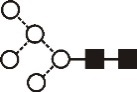 |
| 1493 | M_6_N_2_ | *31.5* | *20.2* | *30.5* | *27.0* | *17.3* | *15.2* | *18.0* | 16.6 | 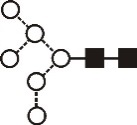 |
| 1534 | M_5_N_3_ | *1.1* | 1.7 | 1.1 | 1.5 | 1.2 | 1.4 | 1.7 | 0.5 | 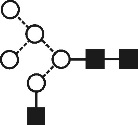 |
| 1559 | M_3_N_4_F_1_ | 0.2 | 0.7 | 0.2 | 0.6 | 3.2 | 2.9 | 1.1 | 6.2 | 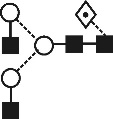 |
| 1655 | M_7_N_2_ | *26.4* | *21.5* | *18.9* | *17.0* | *7.7* | *10.3* | *3.4* | 6.5 | 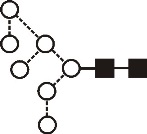 |
| 1696 | G_1_M_5_N_3_ | 1.8 | 2.3 | 1.1 | 2.7 | 4.7 | 3.9 | 5.9 | 5.5 | 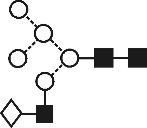 |
| 1721 | G_1_M_3_N_4_F_1_ | - | - | - | - | 3.9 | 3.7 | 6.0 | 7.9 | 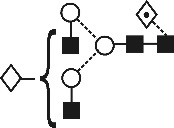 |
| 1737 | G_2_M_3_N_4_ | 0.6 | 0.9 | 1.4 | 1.3 | - | - | - | - | 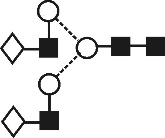 |
| 1817 | M_8_N_2_ | *21.1* | *24.4* | *21.7* | *14.3* | 5.1 | *7.3* | *4.4* | *8.5* | 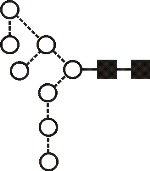 |
| 1842 | G_1_M_5_N_3_F_1_ | 0.3 | 0.6 | 0.4 | 0.5 | 2.1 | 1.7 | 1.7 | 3.4 | 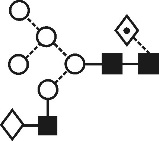 |
| 1858 | G_2_M_5_N3 | 1.1 | 1.0 | 1.4 | 1.5 | 3.1 | 2.1 | 2.1 | 2.1 | 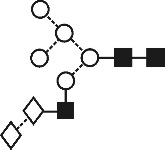 |
| 1883 | G_2_M_3_N_4_F_1_ | *2.1* | *1.2* | *1.8* | 1.6 | *1.6* | *4.1* | *1.3* | *6.7* | 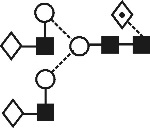 |
| 1899 | G_3_M_3_N_4_ | 0.8 | *1.7* | 1.8 | 2.2 | - | - | - | - | 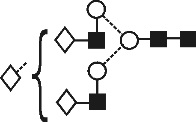 |
| 1940 | G_2_M_3_N_5_ | - | 1.0 | 1.9 | *4.6* | - | - | - | - | 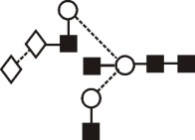 |
| 1979 | M_9_N_2_ | *2.7* | *10.2* | *1.0* | *13.8* | *14.3* | *11.2* | *7.5* | 8.0 | 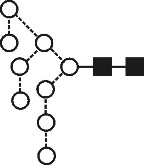 |
| 2029 | G_2_M_3_N_4_F_2_ | 0.6 | 0.3 | 0.7 | 0.4 | - | - | - | - | 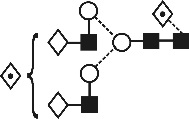 |
| 2045 | G_3_M_3_N_4_F_1_ | *2.9* | *1.9* | *2.5* | 1.2 | 2.0 | 4.1 | 2.3 | 5.2 | 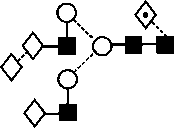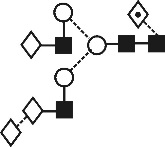 |
| 2061 | G_4_M_3_N_4_ | 0.2 | 0.3 | 0.5 | 0.6 | - | - | - | - | 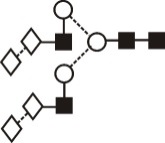 |
| 2086 | G_2_M_3_N_5_F_1_ | 0.9 | 0.8 | 1.4 | 1.6 | 0.8 | 1.9 | 1.4 | 2.1 | 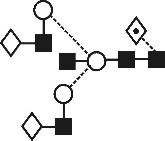 |
| 2102 | G_3_M_3_N_5_ | - | 0.4 | 0.6 | 1.0 | - | - | - | - | 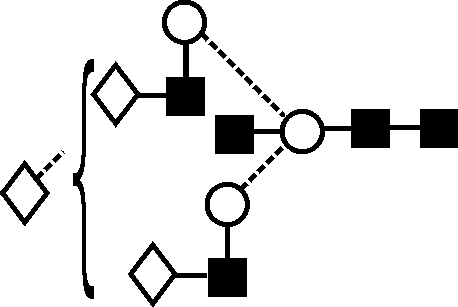 |
| 2141 | Glc_3_M_7_N_2_ | - | - | - | - | *21.1* | *16.7* | *31.0* | 10.4 | 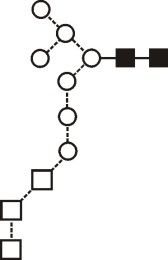 |
| 2191 | G_3_M_3_N_4_F_2_ | 0.5 | 0.5 | 0.6 | 0.2 | - | - | - | - | 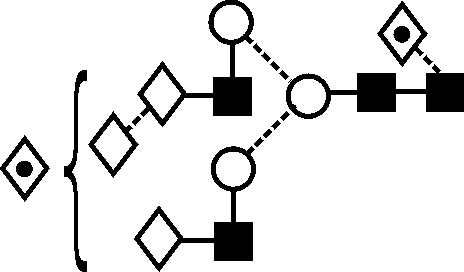 |
| 2207 | G_4_M_3_N_4_F_1_ | *0.7* | 0.3 | *0.9* | 0.4 | 0.8 | 1.1 | 0.8 | 2.5 | 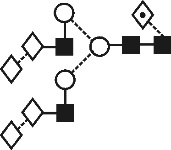 |
| 2232 | G_2_M_3_N_5_F_2_ | 0.7 | 0.5 | 1.5 | 0.5 | - | - | - | - | 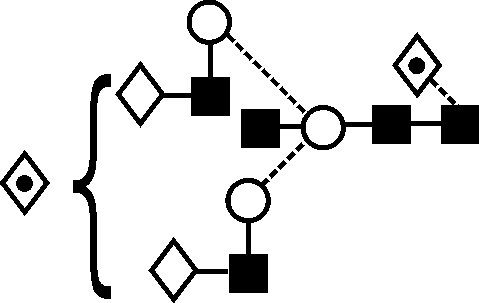 |
| 2248 | G_3_M_3_N_5_F_1_ | *0.5* | *0.7* | *1.3* | 1.0 | - | - | - | - | 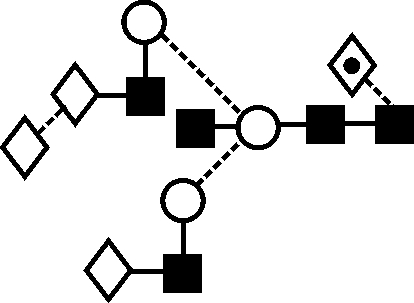 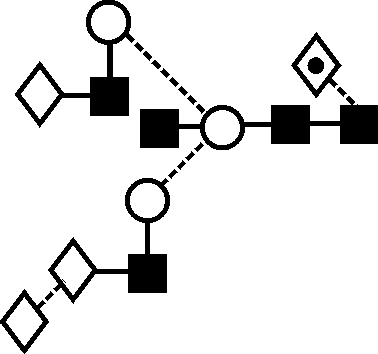 |
| 2303 | Glc_3_M_8_N_2_ | - | - | - | - | *4.9* | 4.4 | *6.2* | 2.7 | 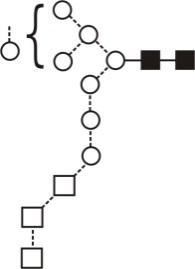 |
| 2394 | G_3_M_3_N_5_F_2_ | 0.4 | 0.6 | 1.2 | 0.4 | - | - | - | - | 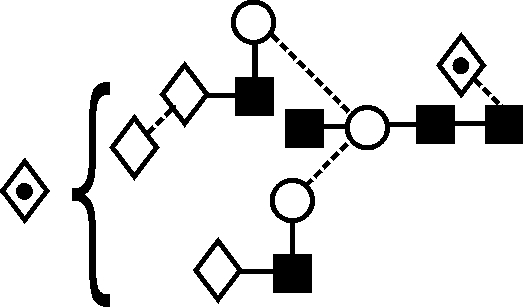 |
| 2410 | G_4_M_3_N_5_F_1_ | 0.4 | 0.3 | 0.4 | 0.3 | - | - | - | - | 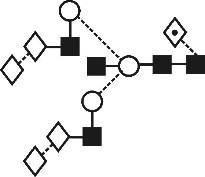 |
| 2465 | Glc_3_M_9_N_2_ | - | - | - | - | 0.4 | 0.7 | 0.1 | 0.2 | 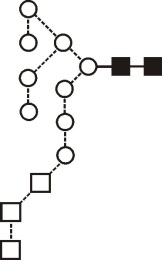 |

**Table S2. Masses, compositions, (proposed) structures and quantitative data for the N-glycans from mass spectrometry analysis of influenza glycoproteins from virus purified from *N*N-DNJ-treated cells.** MDCK cells were infected with Udorn, Brisbane/10 and Lviv and treated with 62.5 µM *N*N-DNJ or the equivalent DMSO control. Virus from cell culture supernatants was purified by ultracentrifugation, proteins from pellets were separated by SDS-PAGE, glycoprotein bands were cut and digested glycans were analysed by mass spectrometry as described in materials and methods. **^a^** Calculated monoisotopic masses. All measured masses were within 0.1 Da of these values. **^b^** Glycans G = galactose, M = mannose, Glc = glucose, N = *N*-acetyl glucosamine , F = fucose. Glycans from Udorn HA, Brisbane/10 HA, Lviv HA and NA are expressed as a percentage of total glycans. **^c^** Compounds for which fragmentation data were obtained are listed in italics. Structures where no fragmentation data are available are based on the composition deduced from the mass and structural features derived from the structures that were confirmed by fragmentation, and from known biosynthetic pathways. **^d^** Symbols for the monosaccharides: mannose (open circle), galactose (open diamond), glucose (open square), *N*-acetyl glucosamine (filled square), fucose (dotted diamond). The angle of the line connecting the symbols shows the linkage (2-link (vertical line), 3-link (forward slash), 4-link (horizontal line), 6-link (back slash)). β-bonds (solid line), α-bonds (dashed line). Full details of the symbolic style used for the glycans is described by Harvey *et al.*.^49^

## **Supplementary material**

**Primer sequences**

IUB codes: **A** (Adenosine), **C** (Cytidine), **G** Guanosine, **T** (Thymidine), **H** (A, C or T), **R** (A or G), **Y** (C or T), **M** (A or C), **W** (A or T), **N** (any base).

For reverse transcription, the **Uni-12** primer: 5’ AGC-AAA-AGC-AGG 3’was used.

Primers for amplification of genomic segments and sequencing are as follows (in the primer name F = forward primer, R= reverse primer):-

*Segment 1:*

*(H3N2/H1N1)* **H5PB2F1** 5’ AGCAAAAGCAGGTCAATTATATTCA 3’,

*(H1N1)* **cswPB2R853** 5’ GGTCTGCTGACACTGCTGCTCTTC 3’,

*(H3N2)* **HPB2R960** 5’ GCATATRTCCACAGCTTGTTCTTC 3’

*Segment 2:*

*(H3N2/H1N1)* **H5PB1F1** 5’ AGCAAAAGCAGGCAAACCATTTG 3’,

*(H1N1)* **cswPB1R844** 5’ CTACTGGGAGCCCAGACTGTTCAAG 3’,

*(H3N2)* **PB1-1262R** 5’ TTRAACATGCCCATCATCAT 3’

*Segment 3:*

*(H1N1)* **cswPAF26** 5’ GGAAGACTTTGTGCGACAATGCTTC 3’,

*(H1N1)* **cswPAR884** 5’ GCAGGAACTTTGACCGCTGATGGC 3’,

*(H3N2)* **HPAF597** 5’ TCGTCAGTCCGAAAGAGGCGAAGA 3’,

*(H3N2)* **PA-1498R** 5’ TNGTYCTRCAYTTGCTTATCAT 3’

*Segment 4:*

*(H1N1)* **H1F1** 5’ AGCAAAAGCAGGGGAAAATAAAAGC 3’,

*(H1N1)* **H1HAR1264** 5’ CCTACTGCTGTGAACTGTGTATTC 3’,

*(H3N2)* **H3HAF6** 5’ AAGCAGGGGATAATTCTATTAACC 3’,

*(H3N2)* **H3A1R1** 5’ GTCTATCATTCCCTCCCAACCATT 3’

*Segment 5:*

*(H3N2/H1N1).* **NPFUc** 5’ TATTCGTCTCAGGGAGCAAAAGCAGGGTWRATAAC *3’,*

*(H1N1)* **cswNPR1202** 5’ CAGTATCTGCTTCTCAGTTCCAGG *3’,*

*(H3N2)* **HNPR1010** 5’ GGATTCTCGTTHGGTCTGATTAGG *3’*

*Segment 6:*

*(H3N2)* **N1F1** 5’ AGCAAAAGCAGGAGTTTAAAATG 3’,

*(H3N2)* **N1R1099** 5’ CCTATCCAAACACCATTGCCGTAT 3’,

*(H1N1)* **NAFUc** 5’ TATTGGTCTCAGGGAGCAAAAGCAGGAGT 3’

*(H3N2)* **N2R1104** 5’ ATCCACACGTCATTTCCATCATCA 3’

*Segment 7:*

*(H3N2/H1N1)* **MF1** 5’ AGCAAAAGCAGGTAGATATTGAAAGA 3’,

*(H3N2/H1N1)* **MR1027** 5’ AGTAGAAACAAGGTAGTTTTTTACTC 3’

*Segment 8:*

*(H3N2/H1N1).* **NSF1 5’** AGCAAAAGCAGGGTGACAAAGACA 3’,

(H1N1) **cswNSR890 5’** AGTAGAAACAAGGGTGTTTTTTAT 3’.

(H3N2) **NSAR641** 5’ GYTTCTCCAAGCGAATCTCTGTA 3’.
